# Time-Resolved Monitoring of the Oxygen Transfer Rate of Chinese Hamster Ovary Cells Provides Insights Into Culture Behavior in Shake Flasks

**DOI:** 10.3389/fbioe.2021.725498

**Published:** 2021-08-25

**Authors:** Nina Ihling, Lara Pauline Munkler, Christoph Berg, Britta Reichenbächer, Johannes Wirth, Dietmar Lang, Roland Wagner, Jochen Büchs

**Affiliations:** ^1^ AVT—Biochemical Engineering, RWTH Aachen University, Aachen, Germany; ^2^ Rentschler Biopharma SE, Laupheim, Germany

**Keywords:** Chinese hamster ovary (CHO cells), mass transfer characteristics, shake flask cultivation, oxygen transfer rate, time-resolved monitoring

## Abstract

Cultivations of mammalian cells are routinely conducted in shake flasks. In contrast to instrumented bioreactors, reliable options for non-invasive, time-resolved monitoring of the culture status in shake flasks are lacking. The Respiration Activity Monitoring Respiration Activity Monitoring System system was used to determine the oxygen transfer rate (OTR) in shake flasks. It was proven that the OTR could be regarded as equal to the oxygen uptake rate as the change of the dissolved oxygen concentration in the liquid phase over time was negligibly small. Thus, monitoring the oxygen transfer rate (OTR) was used to increase the information content from shake flask experiments. The OTR of a Chinese hamster ovary cell line was monitored by applying electrochemical sensors. Glass flasks stoppered with cotton plugs and polycarbonate flasks stoppered with vent-caps were compared in terms of mass transfer characteristics and culture behavior. Similar mass transfer resistances were determined for both sterile closures. The OTR was found to be well reproducible within one experiment (standard deviation <10%). It correlated with changes in cell viability and depletion of carbon sources, thus, giving more profound insights into the cultivation process. Culture behavior in glass and polycarbonate flasks was identical. Monitoring of the OTR was applied to a second culture medium. Media differed in the maximum OTR reached during cultivation and in the time when all carbon sources were depleted. By applying non-invasive, parallelized, time-resolved monitoring of the OTR, the information content and amount of data from shake flask experiments was significantly increased compared to manual sampling and offline analysis. The potential of the technology for early-stage process development was demonstrated.

## Introduction

Complex therapeutic proteins are dominantly produced using mammalian cell cultivation with Chinese hamster ovary (CHO) cells as the preferred host (Wurm, 2004). While manufacturing is usually carried out in large bioreactors, early-stage process development relies on smaller devices of different scales.

Although manufacturers can choose from a broader range of different small-scale cultivation systems for mammalian cell cultivations by now, the information content from these systems is less compared to highly instrumentalized bioreactors (Betts and Baganz, 2006; Bareither and Pollard, 2011). For characterization on a single-cell level, microfluidic culture devices are recently applied for mammalian cells (Schmitz et al., 2021). Microtiter plates can be used for cultivation at an early stage of process development (Girard et al., 2001; Deshpande and Heinzle, 2004). However, the lower the culture volume, the greater the relative evaporation rate. Substantial evaporation is a significant drawback of very small cultivations systems, particularly during long-term cultivations necessary for bioprocess development studies (Betts and Baganz, 2006). In addition to TubeSpin® bioreactors with a volume of 50 ml (Girard et al., 2001; De Jesus et al., 2004; Villiger-Oberbek et al., 2015; Gomez et al., 2017) and the Ambr15® system with a volume of 10–15 ml (Rameez et al., 2014), shake flasks are very often utilized for research issues and early-stage development studies in animal cell culture-based protein, virus, and viral vector production. They enable using appropriate scalable volumes that allow to take several samples of higher volumes for different quality analyses. Further, they are much cheaper and flexible compared to spinner flasks. The latter also have limitations in oxygen supply and mixing, which is particularly disadvantageous for oxygen-demanding insect cells (Annathur et al., 2003).

As the space for sensor integration in and around the culture devices is usually limited, it is crucial to identify the parameter with the highest relevance and information content for a desired application. The identified parameter should preferably be acquired non-invasively to avoid culture contamination and interference with the cultivation process.

For some years, especially measurement of dissolved oxygen and pH, more recently also dissolved CO_2_, has been realized in several small-scale cultivation systems for different mammalian cell lines (Betts and Baganz, 2006; Hanson et al., 2007; Naciri et al., 2008; Vallejos et al., 2012; Ude et al., 2014; Chatterjee et al., 2015). These techniques give time-resolved information on the culture status with a higher resolution than manual sampling. For pH and dissolved oxygen measurement, optodes, which are oxygen or pH-sensitive fluorescent metal complexes immobilized at the bottom of the flask or a microtiter plate, were used (Kostov et al., 2001; Tolosa et al., 2002; Vallejos et al., 2012). Here, the cells get in direct contact with parts of the measurement device, i.e., the optode. In addition, the measurement might be carried out in a sampling loop (Chatterjee et al., 2015). However, this complicates the experimental setup, which is not desirable for routine application. Moreover, the pH is usually kept in a narrow range during cultivation by adjusting with carbon dioxide or synthetic buffers (Li et al., 2010), resulting in limited information content from this parameter.

When it comes to monitoring options, measurement of the oxygen uptake rate (OUR) is particularly suited to determine the culture status of mammalian cells. The OUR is a universal indicator for metabolic activity and culture behavior, as it is linked to the physiological state of aerobic cells (Lecina et al., 2006; Ratledge and Kristiansen, 2008; Doran, 2013). Advances in the application of OUR measurement for bioprocess development of mammalian cells have recently been summarized in a comprehensive review, underlining the impact of OUR as a key parameter for bioprocess monitoring and development (Martinez-Monge et al., 2019).

In contrast to conventional sampling, OUR measurements can be performed with high-frequency and enable real-time adjustments of the process. Thus, OUR measurement was shown to be suited for cell line characterization (Ramirez and Mutharasan, 1990; Deshpande and Heinzle, 2004; Huang et al., 2010; Seidel et al., 2021), the design of nutrient feeding strategies (Kyung et al., 1994; Eyer et al., 1995; Zhou et al., 1997; Goldrick et al., 2018) and estimation of the viable cell concentration (VCC) (Fleischaker and Sinskey, 1981; Yamada et al., 1990; Zhou et al., 1995; Kamen et al., 1996; Higareda et al., 1997; Galvez et al., 2012). In combination with VCC measurement, the OUR can be used to calculate cell-specific oxygen uptake rates (qO_2_), which is a crucial parameter of every cell line (Ruffieux et al., 1998).

A fundamental difference between measurement of the OUR and the dissolved oxygen tension (DOT) lies in the type of information provided by OUR and DOT, respectively. While the DOT reflects the present concentration of oxygen, the OUR contains time-dependent information. Since the DOT’s absolute change is dependent on the process conditions, determined DOT values are not comparable when culture conditions (e.g., filling volume or shaking frequency) are changed. On the other hand, OUR values can be compared directly between different culture conditions, including different cultivation devices and scales. In addition, as introduced above, relations between OUR and other metabolic parameters (VCC etc.) are well established, enabling to derive these parameters from measured OUR data directly.

The Respiration Activity Monitoring System (RAMOS) has been reliably established for microbial research to determine the OTR in shake flasks. RAMOS utilizes electrochemical oxygen sensors to measure the oxygen partial pressure in the headspace of shake flasks (Anderlei and Büchs, 2001; Anderlei et al., 2004). RAMOS is commonly used to monitor eight flasks in parallel, but parallelization is only limited by the number of shake flasks integrated into the incubator. Besides monitoring of rather “strongly metabolizing” bacteria and yeast cells, the applicability of RAMOS has also been demonstrated for cells with a low breathing activity such as a hybridoma cell line (Anderlei et al., 2004) and the microalgae *Chlorella vulgaris* (Keil et al., 2019). Moreover, HEK293 cells have recently been cultured in single-use shake flasks with simultaneous monitoring of the OTR and the carbon dioxide transfer rate (CTR) (Anderlei et al., 2020). Last, monitoring of the respiration activity was combined with pH measurement by applying a pH optode to further increase process insights (Scheidle et al., 2007). When it comes to scalability, it has been demonstrated that results obtained from microbial RAMOS shake flask experiments in batch mode are transferable to microtiter plates as well as stirred tank reactors, if the operating conditions are adjusted properly (Wewetzer et al., 2015). In addition, also results from microbial fed-batch shake flask experiments were demonstrated to be transferable to larger scales (Müller et al., 2019). Furthermore, the scalability of CHO cultures from small orbitally shaken culture vessels to the 1,000 L scale has also been demonstrated (X. Zhang et al., 2010).

The oxygen mass transfer is influenced by two resistances in shake flasks: the sterile closure and the gas-liquid interface. Mass transfer between the gas and liquid phase is usually considered rate-limiting during cultivation (Truesdale et al., 1955). However, depending on the properties of the used sterile closure, the closure can also affect the total oxygen mass transfer (Mrotzek et al., 2001).

The mass transfer resistance between the gas and liquid phase is described by the volumetric oxygen transfer coefficient (k_L_a). Erlenmeyer flasks made from glass have been extensively investigated in terms of gas-liquid mass transfer (Azizan and Büchs, 2017; U.; Maier and Büchs, 2001; Ulrike; Maier et al., 2004; H.; Zhang et al., 2005). For single-use shake flasks made from polycarbonate, which are typically used in mammalian cell culture experiments, gas-liquid mass transfer was also described by k_L_a determination (Schiefelbein et al., 2013). For 250-ml shake flasks using 80 ml working volume, a chemically defined culture medium and 150 rpm shaking speed, k_L_a values between 8 and 15 h^−1^ are reported (Tsai et al., 2012). For 140 rpm and 250 ml flask volume, a k_L_a of 25 h^−1^ is reached with 50 ml filling volume (Schiefelbein et al., 2013). The k_L_a values for glass and polycarbonate flasks were found to be in the same order of magnitude. However, a negative impact of hydrophobic flask material was also reported in the literature (U. Maier and Büchs, 2001). Generally, caution needs to be paid when comparing k_L_a values, as the methods for its determination tend to vary quite strongly (Seidel et al., 2021).

Shake flasks are typically equipped with a sterile closure to keep the interior sterile. However, this sterile closure acts as an additional resistance for oxygen mass transfer. Consequently, the closure also needs to be characterized in terms of mass transfer. For the characterization of mass transfer attributes of sterile closures, a model was previously developed (Henzler and Schedel, 1991). The model considers convective and diffusive gas transfer contributions. Mrotzek et al. (2001) combined the model with measuring the water evaporation rate to determine the mass transfer characteristics for carbon dioxide (CO_2_) and oxygen for different sterile closures. For 250-ml Erlenmeyer flasks stoppered with cotton plugs, the sterile closure’s mass transfer resistance was much smaller than the gas-liquid mass transfer resistance (Mrotzek et al., 2001; Anderlei et al., 2007). Consequently, gas-liquid mass transfer limits the total oxygen supply of cultures in shake flasks. Even though polycarbonate shake flasks with a vent-cap are routinely applied to cultivate mammalian cells, the mass transfer resistance of the vent-cap has not been investigated in detail up to now, to the best of the author’s knowledge.

Accurate and time-resolved knowledge of the OUR and the corresponding derived cellular and metabolic information of CHO cells in parallel shake flasks would represent an important advantage to existing technology, as no fully instrumented and controlled bioreactors would be needed for data acquisition. Thus, information about the OUR in small-scale experiments would enable an easier, more economically and parallelized option to gain valuable insights on the culture status. A non-invasive and simple experimental setup would also represent an important extension to technology already established in shake flasks and other small-scale systems. Consequently, our study demonstrates time-resolved, parallelized, non-invasive monitoring of the OUR in shake flasks for the first time applied to an industrially relevant CHO cell line. The usefulness of the technology is further investigated by comparing two different culture media and utilizing glass as well as single-use polycarbonate flasks.

## Materials and Methods

### Evaporation Measurement

Evaporation from 250-ml Erlenmeyer glass flasks stoppered with cotton plugs (culture plug Rotilabo®) and from 250-ml polycarbonate flasks closed with a vent-cap (Corning® CLS431144 purchased from Sigma Aldrich) was determined gravimetrically. Flasks (*n* = 5 each) were filled with 50 ml deionized water and run under cultivation conditions at a temperature of 36.5°C, 140 rpm shaking speed, 70% relative humidity, 5% CO_2_ at a shaking diameter of 50 mm in an incubator (ISF1-X, Kühner AG, Switzerland). Subsequently, the water evaporation rate was calculated from the decrease in weight over time.

### Producer Cell Line

A Chinese hamster ovary (CHO) cell line producing an IgG monoclonal antibody was used for the experiments. The cell line is Rentschler Biopharma’s proprietary mammalian cell line which is a pre-tagged CHO K1 master cell line for directed integration of the gene of interest by recombinase mediated cassette exchange (RMCE) (Rehberger et al., 2013).

### Adaptation of the RAMOS Device

**FIGURE 1:**
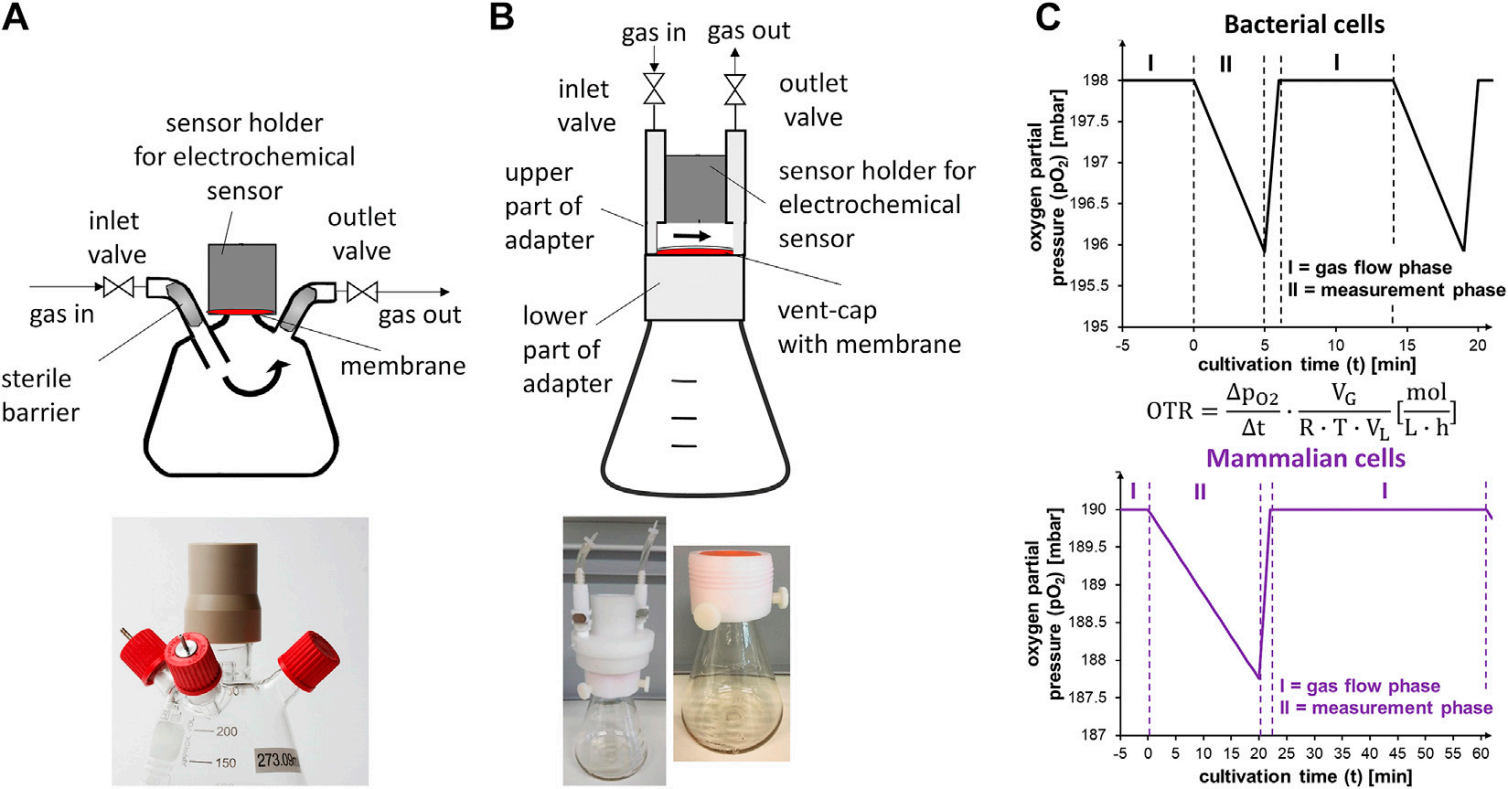
Flasks used for online monitoring of the oxygen transfer rate in this study and schematic change of oxygen partial pressure during cultivation. **(A)** Schematic representation of the gas flow (arrows) and modification of the glass flasks for active aeration and measurement of the OTR **(top)**. The sensor is separated from the headspace of the flask by a gas permeable membrane (red area). Exemplary picture of the modified glass flasks **(bottom)**. **(B)** Schematic representation of the gas flow (arrows) and polycarbonate flasks with mounted adapter (grey) **(top)**. The oxygen sensor is separated from the headspace of the flask by the membrane in the vent-cap (red area). The gap in the adapter is needed for gassing. Gas volume between sensor and membrane is approximately 3 ml. Exemplary pictures of flasks with mounted adapter placed on top **(bottom)**. **(C)** Simplified schematic change of the oxygen partial pressure (pO_2_) during cultivation of bacterial cells **(upper diagram)** and mammalian cells **(lower diagram)** in the RAMOS device. I: gas flow phase (inlet and outlet valves open). II: measurement phase (inlet and outlet valves closed). High-flow phase after measurement phase and cut-off-phase (first part of measurement phase with variable length) are depicted here and explained later in the manuscript. Equation in the middle shows calculation of the oxygen transfer rate (OTR) (mol L^−1^ h^−1^) from the change of oxygen partial pressure over time ($\frac{\Delta {\text{pO}}_{2}}{\text{dt}}$) (bar h^−1^), gas volume of the flask V_G_ [L], liquid volume of the flask V_L_ [L], universal gas constant R (bar L mol^−1^ K^−1^), and temperature T [K].

A blueprint of the RAMOS device as described by Anderlei et al. was adapted for cultivation of CHO cells (Anderlei and Büchs, 2001; Anderlei et al., 2004). Commercial versions of the RAMOS device are available from HiTec Zang GmbH (Herzogenrath, Germany) and Kühner AG (Birsfelden, Switzerland). Calculated OTR values deviating more than 15% (medium 1) or 30% (medium 2) from the previous and subsequent calculated OTR values were considered outliers and not further regarded. Deviations are caused, if the incubator hood is opened just before or during the measurement phase, as this affects the temperature inside the incubator. Additionally, as the sensor is mounted right on top of the flask (see Figure 1), the cable connecting the sensor to the measurement unit is subjected to mechanical stress, which might also cause misreadings. Additional details on the adaptation of the device are given in *Principle and Adaptation of the RAMOS Device*.

### Cultivation Conditions

All cultivations were carried out in 250-ml flasks with 50 ml filling volume, being shaken on an orbital shaker with a 50 mm shaking diameter. The shaking frequency was set to 140 rpm and the temperature to 36.5°C. Flasks for passaging and manual sampling with subsequent offline analysis were cultured in an incubator (ISF1-X, Kühner AG, Switzerland) at a controlled relative humidity of 70% and a controlled CO_2_ concentration of 5%, respectively. Flasks for time-resolved monitoring of the OTR were operated in parallel in an incubator (ISF1-X, Kühner AG, Switzerland) under the same conditions but without CO_2_ and humidity control. Instead, these flasks were directly gassed with a gas mixture of 5% CO_2_ in synthetic air (19.5% O_2_) from a gas bottle (Figure 1A). Cultivations were either carried out in PowerCHO^TM^ two serum-free chemically defined culture medium, containing HEPES buffer and Pluronic® F68 (Lonza AG, Switzerland) (“medium 1”) or in EX-CELL® Advanced™ CHO Fed-batch Medium (Sigma-Aldrich, United States) (“medium 2”). Both culture media were supplemented with 6 mM L-glutamine from a 200 mM stock solution (Gibco Life Sciences, Thermo Fisher Scientific, United States) before cultivation. Directly before use, culture media and supplements were pre-heated to 36.5°C for about 30 min in a water bath (VWB2 12, VWR, USA).

### Working Cell Bank and Cryopreservation

A working cell bank (WCB) with vials containing a viable cell concentration (VCC) of 1.2∙10^7^ cells mL^−1^ was stored in the vapor phase of liquid nitrogen. For that, cells were resuspended in a mixture of spent culture medium (45% v/v), fresh culture medium (45% v/v and dimethylsulfoxide (DMSO) (10% v/v) (Sigma-Aldrich, United States). 1 ml of the cell suspension was frozen at −80°C in 2 ml cryogenic vials for 24 h with a controlled cooling rate of −1°C min^−1^ using the Nalgene Mr. Frosty^TM^ freezing container (Thermo Fisher Scientific, United States) according to manufacturer’s protocol.

### Cell Passaging

Before cultivation, one vial stored in liquid nitrogen was rapidly thawed, and cells resuspended in 9 ml pre-heated culture medium. Subsequently, the cells were centrifuged for 10 min at 150 × *g* and at room temperature in a Heraeus Multifuge X3R centrifuge (Thermo Fisher Scientific, United States). The remaining cell pellet was resuspended in 10 ml pre-heated medium for cultivation of the first cell passage. After 72 h, 40 ml of fresh medium were added to the flask. The cell culture was split every three to 4 days. The seed cell concentration was set to 0.2∙10^6^ cells mL^−1^ (3-days split) or 0.1 ⋅ 10^6^ cells mL^−1^ (4-days split) in a cultivation volume of 50 ml. For all steps, 250 ml Corning® polycarbonate Erlenmeyer flasks with vent-cap (Sigma-Aldrich, United States) were used.

### Main Culture Experiments

An overview of the experiments presented in this study is depicted in Supplementary Table S1.

After a number of passages, as shown in Supplementary Table S1, the main culture was started at a seed cell concentration of 0.2∙10^6^ cells mL^−1^. PenStrep (stock with 10,000 Units mL^−1^ penicillin and 10 g L^−1^ streptomycin, Sigma-Aldrich, United States) or kanamycin (stock with 30 g L^−1^, Carl Roth, Germany) was added to the culture, resulting in a concentration of 1% or 30 mg L^−1^, respectively, for the final culture medium. Cultivation was carried out in 250-ml Corning® polycarbonate flasks with vent-cap (“single-use polycarbonate”) (Sigma-Aldrich, United States) or 250 ml Erlenmeyer glass flasks (Duran, Schott AG, Germany).

### Sampling

At each sampling point, 1.5 ml of culture broth were manually sampled under sterile conditions from the “offline” flasks. As offline flasks, either glass flasks stoppered with a cotton plug or polycarbonate flasks with vent-cap (see above) were used. After sampling, the flasks were shaken up to the next sampling point and until the end of the cultivation. Viable and total cell concentration, pH value, and osmolality were determined directly after sampling, as stated below. For further analysis, the supernatant was stored at −20°C until used.

### Determination of Offline Parameters

#### Viable and Total Cell Concentration

Viable and total cell concentrations were determined by manual cell counting with a hemocytometer (Counting chamber C-Chip Neubauer improved, Carl Roth, Germany) using the trypan blue staining method (Sigma-Aldrich, United States) according to the manufacturer’s protocol.

During each sampling 1.5 ml culture broth was harvested by centrifugation in a table centrifuge (mini centrifuge Rotilabo, Carl Roth, Germany) for 3 min at a rotation rate of 6,000 rpm at room temperature. The supernatant was stored at −20°C until use for subsequent analysis. The final samples taken from the flasks at the end of the cultivation were centrifuged for 15 min at 22°C and at 225 × g (Heraeus Multifuge X3R, Thermo Fisher Scientific, United States).

#### pH Value

The pH value was determined at room temperature with an InLab Easy pH electrode (Mettler Toledo, Germany) using a CyberScan pH 510 m (Eutech Instruments, Thermo Scientific, Germany). Appropriate buffers at pH four and pH seven were used for a two-point calibration. It should be noted that the measurement of the pH under ambient air conditions will result in higher pH values than under physiological conditions as CO_2_ quickly gasses out, resulting in a rise in pH.

#### Glucose and Lactate

The concentrations of glucose and lactate were determined by HPLC using a Dionex Ultimate 3,000 system (Thermo Scientific, United States). An organic acid resin column (Rezex ROA-Organic Acid H^+^ (8%), 300 × 7.8 mm, Phenomenex Inc., United States) was used for separation at a temperature of 40°C and a flow rate of 0.8 ml min^−1^. As mobile phase, 5 mM H_2_SO_4_ was used. Separation was performed in isocratic mode. A refractive index detector (RefractoMax 520, Shodex, Germany) was used for detection.

#### Osmolality

The osmolality of the supernatant was determined using a freezing point osmometer (Gonotec Osmomat 3,000, Gonotec GmbH, Germany). A three-point calibration with distilled water and standard solutions with a given osmolality (100 and 500 mOsm kg^−1^, respectively), was carried out before measurement.

## Results and Discussion

### Principle and Adaptation of the RAMOS Device

For measurement of the OTR, electrochemical sensors were used (Anderlei and Büchs, 2001). To demonstrate and enable the broad application of monitoring the breathing activity of CHO cells, glass as well as single-use polycarbonate flasks were considered in this study. Differences between both flask types are given in Supplementary Table S2. For glass flasks, specialized flasks with customized inlet and outlet connectors were used and the sensor was mounted using a screwable holder that contained a gas-permeable membrane (Figure 1A). The sensor was placed behind the gas-permeable membrane to keep the interior of the flask sterile. In contrast to glass flasks, polycarbonate (“single-use”) flasks were not intended for multiple use. Further, as the flasks were delivered in sterilized format, a modification of the flasks with a screwable holder and connectors was impractical. Consequently, the electrochemical sensor was placed above the vent-cap using commercially available adapters (Kühner AG, Switzerland) (Figure 1B).

The electrochemical sensors changed their voltage depending on the oxygen partial pressure (Figure 1C). The flask was aerated with a constant volumetric flow during the gas flow phase (Figure 1C, phase I). During the measurement phase (Figure 1C, phase II), the inlet and outlet valves of the measurement flask were closed. As the cells continued to consume oxygen while the valves were closed, the partial pressure in the headspace decreased over time (Anderlei and Büchs, 2001). This decrease was used to calculate the OTR (Figure 1C, equation). For rather high-breathing bacterial cultures, the decrease in partial pressure over time was comparably strong (0.5 mbar min^−1^ at an OTR of about 25 mmol L^−1^ h^−1^ and 10 ml filling volume) (Figure 1C, black curve). Consequently, the measurement time in *E. coli* cultivations, for example, is set to 5 min. The duration of the measurement and gas flow phase can easily be adjusted to the expected respiration activity of the cells, which offers flexibility for monitoring and enables adjustment of the signal quality. For low-breathing mammalian cell cultures (0.1 mbar min^−1^ at an OTR of about 1 mmol L^−1^ h^−1^ and 50 ml filling volume), the measurement phase must be extended to reach an acceptable resolution (Figure 1C, purple curve) as reported for the cultivation of HEK293 cells, where a measuring time of 20–30 min was recommended (Anderlei et al., 2020). A comparably short (1–2 min) “high-flow” phase (not shown) followed the measurement phase to quickly increase the partial pressure of oxygen inside the flask up to its level before the measurement phase. The high-flow phase ensured that repeated measurement phases did not result in a progressing decrease of the partial pressure of oxygen inside the shake flask. The high-flow phase was followed by a gas flow phase (phase II) that was usually twice as long as the measurement phase.

As a result of the different breathing activities, OTR determination by RAMOS might be carried out with a frequency of four measurement points per hour for microbial cultivations. For a typical bacterial batch cultivation that lasts 24 h, the number of data points would be 96 (one measurement value every 15 min). To achieve a comparable number of data points in relation to the overall cultivation time of a mammalian cell culture in batch mode that lasts 4–7 days, one measurement point is required every 60–120 min. Here, the gas flow phase was set to 40 min, and the measurement phase was set to 20 min, to record one measurement point every 60 min. Compared to a continuous “on-line” OTR acquisition, measurement in an interval of every 60 min is not continuous and therefore referred to as “time-resolved”.

### Differences in Gas Delivery and Mass Transfer Resistance of the Sterile Closure

Glass flasks in the RAMOS device were actively gassed (Figure 1A). The gassing rate chosen needed to correspond to the mass transfer resistance of the sterile closure (Klöckner et al., 2013). Choosing an appropriate gassing rate ensured that the gas headspace concentrations were comparable for “online” and “offline” flasks, which is a prerequisite for validly comparing them with data from manual sampling. A gassing rate of 10 ml min^−1^ was previously determined to result in comparable gas headspace concentrations between actively aerated glass flasks in the RAMOS device and glass flasks stoppered with a cotton plug (Klöckner et al., 2013). However, the mass transfer characteristics, represented by the mass transfer resistance for oxygen (k_st_,_O2_), of the exact cotton plugs used in this study were not previously determined.

The adapters placed on top of the single-use flasks allowed a defined gas flow above the vent-cap (Figure 1B, arrows). For this purpose, the upper part of the adapter had a gap between the sensor and the membrane (Figure 1B). Compared to the modified glass flasks of the conventional RAMOS system (Figure 1A), the type of aeration had to be considered when mass transfer phenomena in the two flask types were described. Further, the mass transfer resistance of vent-caps was not quantitatively described previously. Since the sterile closures (cotton plug and vent-cap) differed significantly in material and design, k_st_,_O2_ of the polycarbonate flasks with vent-caps was investigated.

The mass transfer characteristics of both sterile closures were determined as described in Supplementary Data *Determination of Mass Transfer Characteristics for Glass and Single-Use Polycarbonate Flasks* (see also Supplementary Figure S1 and Supplementary Table S3). For the cotton plug, a value for k_st_,_O2_ of 0.81∙10^−5^ mol s^−1^ was determined. This value was slightly lower than the values published previously. For 500-ml narrow-necked flasks, values for k_st_,_O2_ of 0.9–1∙10^−5^ mol s^−1^ were determined for individually wrapped cotton plugs, depending on size and bulk density (Mrotzek et al., 2001; Anderlei et al., 2007). Differences between the cotton plugs (made from cellucotton) used in this study and values determined previously could be related to differences in material, density, and fabrication of the plugs and, thus, appear reasonable. For the vent-cap of the single-use polycarbonate flask, k_st_,_O2_ was determined to 1.17⋅10^−5^ mol s^−1^. This value aligns well with the wide- and narrow-necked flasks stoppered with cotton plugs described by Mrotzek et al. (2001). Additionally, Schiefelbein et al. (2013) compared k_L_a values in 500 ml disposable shake flasks and found no difference with and without the cap even though high k_L_a values (∼290 h^−1^) were reached. This observation underlined that the cap of the single-use flask did not impair the total gas transfer even at high gas-liquid mass transfer rates. Also, the permeability of filter caps for 50 ml centrifugation tubes (TubeSpin® reactors, Techno Plastic Products, Trasadingen, Switzerland) was determined (Jordan et al., 2005). These filter tubes had five vent holes of different sizes integrated in the cap. One or more holes could be left open during cultivation to adjust the gas exchange rates. The filter tubes supported cell growth at a filling volume of 30 ml with a volume loss by evaporation of 0.5 g in 5 days (Jordan et al., 2005). Water evaporation from the polycarbonate flasks closed with a vent-cap was 0.017 g h^−1^, which is about 2 g in 5 days. This evaporation rate is comparable with filter tubes when all ventilation holes were open and four times larger than needed for a filling volume of 30 ml (Jordan et al., 2005). In addition, the gas exchange associated with a loss of water of 2 ml (≈2 g) in 5 days was described to be exceeding the need for mammalian cells (Jordan et al., 2005).

Conclusively, no significant oxygen mass transfer limitation by either sterile closure was expected, and ventilation was assumed to be comparable in glass and polycarbonate flasks under the experimental conditions applied in this study. As gas-liquid mass transfer was also demonstrated to be in the same order of magnitude (Schiefelbein et al., 2013), the maximum OTR (OTR_max_) achievable was expected to be similar between glass and polycarbonate flasks. Quantitative values for OTR_max_ under the applied process conditions were calculated considering the mass transfer resistance between the gas-liquid interface and the sterile closure (Eq. 1) (Amoabediny and Büchs, 2007; Sieben et al., 2016). The OTR_max_ represents the maximal OTR that is theoretically achievable for a given set of cultivation conditions. Consequently, the driving concentration gradient between the oxygen concentration in the liquid (y_O2_, _liquid_) and the molar fraction of oxygen in the gas phase (y_O2_, _outside_) was assumed to be at the maximum. As a result, y_O2,liquid_ was assumed to be zero.

$$\mathrm{OTR}=\;\frac{k_{\text{L}}a\cdot p_{\text{abs}}\cdot L_{\text{O}2}\cdot \left(y_{\text{O}2,\text{outside}}-y_{\text{O}2,\text{liquid}}\right)}{1+\frac{{\text{k}}_{\text{L}}a\cdot p_{\text{abs}}\cdot L_{\text{O}2}\cdot V_{\text{L}}}{k_{\text{st},\text{O}2}}}$$ (1)

A value of 7.11 mmol L^−1^ h^−1^ was obtained for glass flasks closed with a cotton plug. For the single-use flask closed with a vent-cap an OTR_max_ of 7.26 mmol L^−1^ h^−1^ was calculated. Additional values used for this calculation are listed in Supplementary Table S3. In both cases, the mass transfer resistance of the sterile closure (denominator) was much smaller than the resistance of the gas-liquid interface (numerator).

With the lower mass transfer resistance determined for the cotton plugs in this study compared to previously used plugs, a gassing rate of 9 ml min^−1^ (instead of 10 ml min^−1^) was needed for active gassing of online flasks in the RAMOS device. Active gassing from a gas bottle with 5% CO_2_ in synthetic air resulted in an oxygen fraction of 0.195. On the other side, flasks for manual sampling were closed with a cotton plug and passively ventilated in a humidified environment. This resulted in an oxygen fraction of about 0.191. Thus, to achieve comparable headspace concentrations, the gassing rate of the actively aerated flask had to be lowered further. A gassing rate of 6.25 ml min^−1^ was chosen. In contrast to online monitored glass flasks, the online monitored polycarbonate flasks were not actively gassed (Figure 1B). Consequently, the ventilation rate had to be high enough to ensure a constant gas composition in the adapter. Thus, for single-use polycarbonate flasks, the same aeration rate (6.25 ml min^−1^) as for glass flasks was chosen, further enabling the cultivation of glass and polycarbonate flasks in parallel.

**FIGURE 2:**
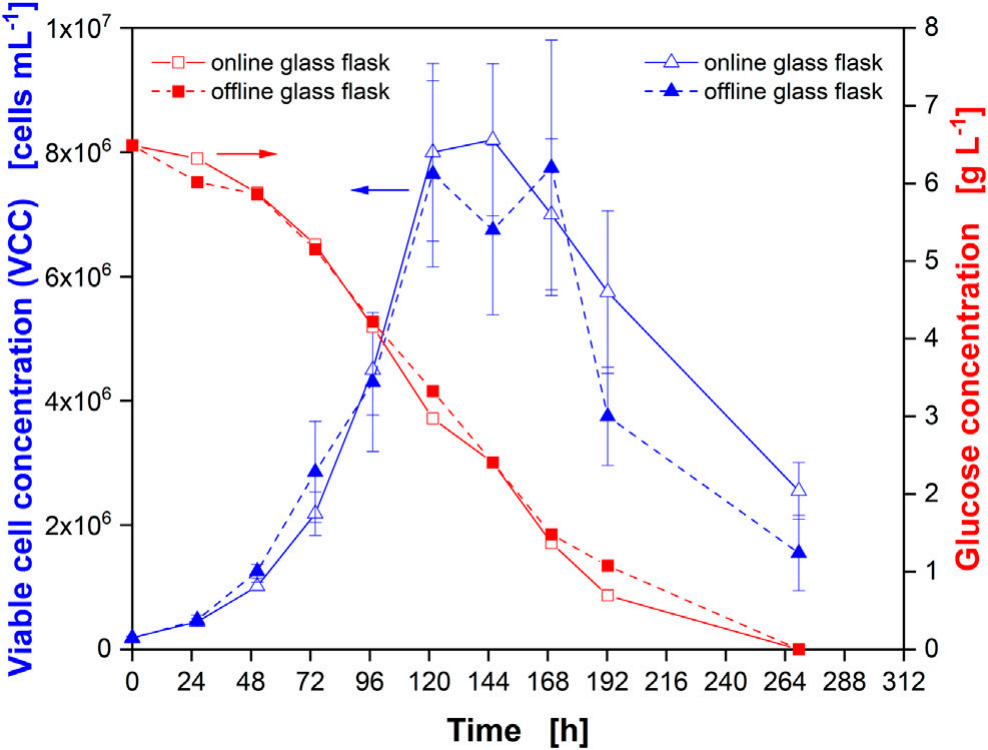
Comparison of Chinese hamster ovary (CHO) cultures in monitored (online) and non-monitored (offline) glass flasks. Viable cell concentration (VCC) in online (open blue triangles) and offline (closed blue triangles) glass flasks as well as glucose concentration in online (open red squares) and offline (closed red squares) flasks. Glass flasks were actively gassed with 5% CO_2_ in synthetic air at a rate of 6.25 ml min^−1^ (online) and passively ventilated by a cotton plug in an incubator environment with 5% CO_2_ in air at 70% RH (offline). Data from experiment 1 (Supplementary Table S1).

To confirm the comparability of the cultivation conditions in actively aerated “online” and passively ventilated “offline” glass flasks, cultivation with CHO cells was simultaneously performed (Figure 2) in both flasks. Samples were directly taken from one online and one offline flask. Similar cell numbers (Figure 2, blue triangles) and glucose consumption (Figure 2, red squares) were observed. During the first part of the cultivation, until the VCC started to decrease (after about 120 h), the data were well aligned, confirming the equal culture behavior for online monitored and offline sampled flasks. In the later stages of the cultivation, the VCC exhibited a large standard deviation, which prohibits a statement on the significance of the difference in viable cell counts between online and offline flasks. However, the measured glucose concentrations still aligned well.

### Cultivation of CHO Cells in Glass Flasks

**FIGURE 3:**
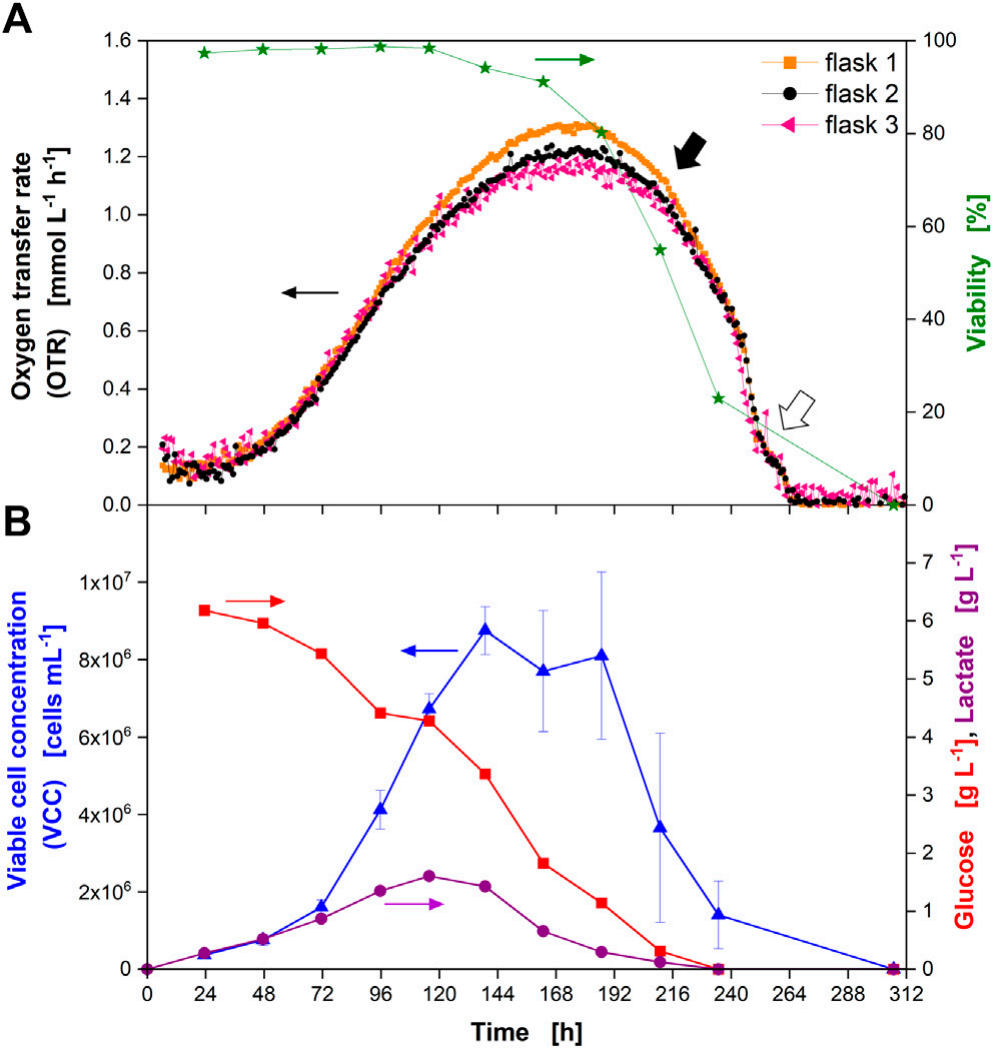
Cultivation of Chinese hamster ovary (CHO) cells in glass shake flasks with time-resolved monitoring of the oxygen transfer rate (OTR) and manual (“offline”) sampling for determination of culture parameters. **(A)** OTR in triplicate (closed orange squares, closed black circles, closed pink triangles) and cell viability (closed green stars) over the cultivation time. **(B)** Viable cell concentration (VCC) (closed blue triangles), glucose concentration (closed red squares), and lactate concentration (closed purple circles) over the cultivation time. Closed black arrow indicates decrease in OTR upon depletion of glucose and lactate, open arrow indicates “shoulder” in OTR. Data from experiment 2 (Supplementary Table S1).

CHO cells were cultivated in triplicate in the adapted RAMOS device using glass flasks. The course of the OTR over the cultivation time for each flask is depicted in Figure 3A. Prior to discussing the data, it first had to be proven that the OTR values determined reflected the OUR of the culture. This is a key prerequisite to utilizing OTR measurement in shake flasks to draw conclusions on culture behavior. As the inlet gas composition and the shaking frequency were kept constant during the experiments, but the OTR changed (see Figure 3A), the dissolved oxygen concentration in the liquid $\left(\frac{{\text{dc}}_{\text{O}2}}{\text{dt}}\right)$ over time also changed. Only if $\frac{{\text{dc}}_{\text{O}2}}{\text{dt}}$ is negligibly small in comparison to the OTR and OUR, the assumption that the OTR is very close to the OUR is justified (Eq. 2).

$$\frac{dc_{\text{O}2}}{\mathrm{dt}}\left[\frac{\mathrm{mmol}}{L\cdot h}\right]=\mathrm{OTR}\;\left[\frac{\mathrm{mmol}}{L\cdot h}\right]-\mathrm{OUR}\left[\frac{\mathrm{mmol}}{L\cdot h}\right]\;$$ (2)

To prove that $\frac{{\text{dc}}_{\text{O}2}}{\text{dt}}$ was indeed negligibly small relative to OTR and OUR, the point with the highest change in the OTR was determined in a worst case scenario. The highest change in the OTR was calculated as the first derivative of the OTR curve (Supplementary Figure S2). After 246 h, the first derivative showed a minimum and highest absolute value. At this point, the OTR changed from 0.44 mmol L^−1^ h^−1^ to 0.38 mmol L^−1^ h^−1^ (ΔOTR = 0.06 mmol L^−1^ in 1 h). From these data, the oxygen concentration in the liquid (c_O2_,_l_) was calculated (Eq. 3):

$${\text{c}}_{\text{O}2,\text{l}}={\text{c}}_{\text{O}2}^{\text{*}}-\frac{\text{OTR}}{{\text{k}}_{\text{L}}\text{a}}$$ (3)

At saturation conditions with air, about 0.22 mmol L^−1^ of oxygen are soluble in water at 36.5°C (Truesdale et al., 1955). The k_L_a was calculated to 42 h^−1^ (Supplementary Table) and the OTR was measured. The change in OTR resulted in a $\frac{{\text{dc}}_{\text{O}2}}{\text{dt}}$ of 0.001 mmol L^−1^ in 1 h, which is roughly 0.45% of the saturation concentration of oxygen in the liquid and much smaller than the OTR. As the measurement phase was 20 min and the oxygen partial pressure in the headspace only decreased by a maximum of 2 mbar during the measurement phase, $\frac{{\text{dc}}_{\text{O}2}}{\text{dt}}$ during the measurement phase could also be neglected. Consequently, $\frac{{\text{dc}}_{\text{O}2}}{\text{dt}}\;$was assumed to be close to zero during the entire cultivation proving that OTR equals OUR to be justified. However, the validity of this assumption has to be reassessed, if the cultivation conditions are changed or if more rapid changes (e.g., substrate depletion at higher cell concentrations) occur. In this case, the magnitude of the error will depend on the specific OUR value and on the k_L_a. Nevertheless, also for fast-growing bacterial cultures (*E. coli*) the mean change in the oxygen concentration was comparably low (∼0.02 mmol L^−1^ h^−1^) (Mühlmann et al., 2018).

The relationship between the OUR and other metabolic parameters (e.g., VCC) has been established for different mammalian cell lines for decades (see Introduction). Thus, this study did not aim to discuss and explain the general progression of the OUR over time. Instead, the possibilities for non-invasive, parallelized monitoring of the OTR in shake flasks should be explored and obtained data validated by comparison with offline samples. As a result, the VCC, viability, glucose and lactate concentration as well as pH and osmolality were determined by manual sampling in parallel to OTR acquisition.

As the OTR was the measuring parameter, the term OTR will be used from here on, even if the OUR was the real physiological variable evaluated. The OTR increased until about 175 h of cultivation (Figure 3A). At this point, an average maximum OTR of about 1.23 mmol L^−1^ h^−1^ was reached. Afterwards, the OTR decreased with a steep drop occurring after 216 h (closed black arrow) and a visible “shoulder” between 253 and 264 h (open arrow). The OTR dropped to zero after 264 h. Even though the measured OTR was low compared to microbial cultivations, the reproducibility within one experiment was high, as seen from the triplicate measurement. The average standard deviation between three independent measurements in one experiment was about 6.5% (until 265 h of cultivation) providing an appropriate reproducibility of the measurement technique. It also proved a reproducible culture behavior in different shake flasks. The value of the standard deviation might be attributed to the slightly higher OTR values reached by the culture in flask 1 (Figure 3A, closed orange squares) and by the slightly fluctuating values obtained from flask 3 (Figure 3A, closed pink triangles). Comparing the maximum OTR reached during cultivation (1.23 mmol L^−1^ h^−1^) to the maximum OTR reachable under the applied shaking conditions (7.11 mmol L^−1^ h^−1^) proved that the cells were not close to an oxygen limitation during cultivation. In addition, the maximum biological OTR measured (1.23 mmol L^−1^ h^−1^) resulted in a decrease of the oxygen partial pressure in the headspace of the flasks of about 2 mbar during the measurement phase (see equation in Figure 1C and Supplementary Table S3 for parameter values). This decrease was very small compared to the oxygen partial pressure inside the flask (∼197 mbar in fully water-saturated air). Consequently, as the headspace gas volume is mixed by the rotating bulk liquid (Anderlei et al., 2007), oxygen gradients inside the flask were not expected. Thus, online monitoring of the OTR was suited to evaluate if the chosen cultivation conditions resulted in a sufficient supply with oxygen.

In parallel to the measurement of the OTR, one flask stoppered with a cotton plug was used for sampling and “offline” determination of culture parameters. The cell viability started to decrease after around 144 h (Figure 3A, closed green stars) and reached zero after 312 h. However, considering the progress of the VCC, it was most likely that the viability reached zero already after about 264 h. In this experiment, kanamycin was supplemented to the medium (see Supplementary Table S1). Kanamycin disrupts bacterial membranes and binds to the cellular membrane of mammalian cells (John et al., 2017). As shown, the viability was above 97% for the first 115 h of the cultivation, indicating that the addition of kanamycin did not affect the viability measurement by trypan blue exclusion.

The VCC increased until about 140 h (Figure 3B, closed blue triangles). Afterwards, it started to decrease (considering the error bars) (see Figure 2, open blue triangles). Cell counts after 187 and 210 h exhibited a large standard deviation (21 and 46%, respectively) due to clumping of the cells (data not shown), which made manual counting error prone.

During the first part of the cultivation, glucose was consumed (Figure 3B, closed red squares) while lactate (Figure 3B, closed purple circles) was produced in parallel until around 120 h of cultivation. Consequently, the pH value decreased (Supplementary Figure S3, open blue squares). After about 120 h, glucose and lactate were consumed in parallel (Figure 3B) and the pH rose again (Supplementary Figure S3, open blue squares). It has to be noted that the pH was determined by manual sampling and not *in-situ*. Due to manual sampling and subsequent pH measurement in ambient air, the absolute values of the pH measured were most likely different from those experienced by the culture that was cultivated in a CO_2_-enriched atmosphere. Nevertheless, the trend of the pH value corresponded well to phases of lactate production and consumption. After 238 h, neither glucose nor lactate were detected in the supernatant, indicating their full depletion. The osmolality steadily decreased during cultivation (Supplementary Figure S3, closed orange circles).

Comparing the shape of the OTR curve with the offline measured data, different conclusions can be drawn directly from the OTR signal. Firstly, the progress of the VCC was reflected by the OTR until the maximum VCC was reached. This was expected since oxygen consumption is an indicator for metabolically active cells. Secondly, the maximum in the OTR was reached after around 180 h, even though glucose was still present in the medium. At this point, the cells had also switched from lactate production to lactate consumption. Thirdly, a rapid decrease in the OTR was observed as soon as glucose and lactate were depleted after about 216 h of cultivation (closed black arrow). This indicated the cells quickly died as soon as these substrates were depleted. This observation is in agreement with data gathered in fully instrumented bioreactors: A rapid decrease of the OUR was attributed to nutrient depletion in fed-batch cultures of NS0 cells and used to adjust feeding time points to increase monoclonal antibody titers (Zhou et al., 1997). For another hybridoma cell line, a shift in the OUR could be attributed to glutamine depletion (Ramirez and Mutharasan, 1990). Consequently, the depletion of major carbon sources could be predicted from the OTR signal.

The rapid decrease of the OTR was interrupted by a visible “shoulder” between 258 and 264 h (open arrow). This on-going metabolic activity was in agreement with the course of VCC and viability, which had not reached zero at this point where glucose and lactate were consumed. This kind of shoulder had been observed for bacterial and yeast cultures in the past (Hansen et al., 2012). In microbial cultures, this “shoulder” is an indicative of a diauxic effect caused by a switch from consumption of one carbon source to another (Hansen et al., 2012). As it is difficult to compare microbial cultures to immortalized cells derived from a mammalian tissue, the reason for the shoulder observed for the CHO culture is currently unknown but could be subject to further studies. For hybridoma cells a similar “shoulder” was seen in the OUR after glutamine depletion, indicating oxidation of alternative substrates (e.g., glucose, other amino acids or fatty acids) (Ramirez and Mutharasan, 1990). As the “shoulder” occurred at the very end of the cultivation, it is less relevant in terms of production. Lastly, the end of active respiration after 264 h could clearly be detected in the OTR signal.

It can be concluded that the observed course of the OTR corresponds well with the offline-determined parameters and is in line with observations made for mammalian cells previously (Ramirez and Mutharasan, 1990; Zhou et al., 1997). In addition, the standard deviation (<6.5%) was relatively low, compared for example to manual cell counting. Consequently, the reliability of the measurement technique was validated. In comparison to results obtained from fully instrumented bioreactors that were utilized for example by Ramirez and Mutharasan (1990), the filling volume was reduced 12-fold. Additionally, data acquisition can be performed e.g., in eight parallelized shake flasks. Compared to offline sampling, the data density was increased 24-fold. However, compared to continuous “on-line” data that can be provided by fully instrumented bioreactors, as already demonstrated decades ago by Ramirez and Mutharasan (1990) and Higareda et al. (1997), the resolution using RAMOS is lower. For metabolic changes occurring faster than the measurement interval, the methodology presented in this study, thus, is not suited. However, depending on the culture conditions and cell-specific oxygen up-take rate of the utilized cell line, the measurement frequency could be increased by decreasing the duration of the measurement phase. As discussed above, a measurement frequency of one measurement value every 60 min is comparable to the measurement resolution of bacterial cultures. Nevertheless, it needs to be pointed out that a truly continuous data acquisition is not possible, as an alternating measurement phase and a gas flow phase are required.

### Cultivation of CHO Cells in Single-Use Polycarbonate Flasks

In parallel to cultivations in glass flasks, a comparable cultivation was carried out in single-use polycarbonate flasks. Cultivation in single-use polycarbonate flasks was carried out to demonstrate flexibility of the monitoring technology. In addition, even though no difference was expected in terms of mass transfer (see above), an influence of the flask material on culture behaviour could not be excluded. It was demonstrated previously that bisphenol A leaching from polycarbonate flasks affected metabolic rates (including glucose consumption and lactate production) of a CHO cell line (Stiefel et al., 2016).

Cultivation in single-use polycarbonate flasks was highly reproducible between the three cultures (average standard deviation in the OTR of about 8%). However, an off-set was observed (Supplementary Figure S4, open orange squares, open pink triangles, open black circles) in all three flasks after the viability (Supplementary Figure S4, open green stars) had dropped to zero. This off-set was not observed during cultivation in glass flasks (Figure 3).

The off-set in the OTR most likely resulted from water vapor diffusion from the inside of the flask through the membrane into the vent-cap to the oxygen sensor in the adapter. Consequently, an off-set was only observed in single-use polycarbonate but not in glass flasks, if the flasks were only filled with water (Supplementary Figure S5). The influence of water vapor diffusion in single-use polycarbonate flasks is further discussed within the Supplementary Data *Influence of Humidity*. As a result of water vapor diffusion, the so-called “cut-off phase” was set to 8 min in this study (see Supplementary Data for a detailed explanation).

**FIGURE 4:**
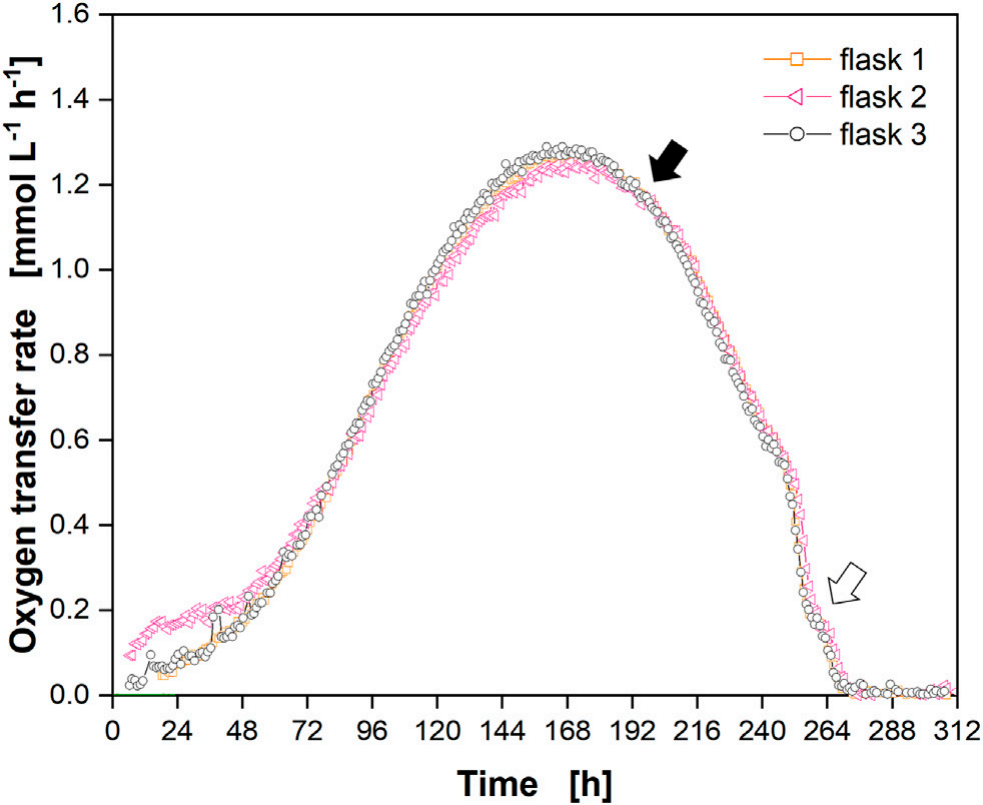
Cultivation of Chinese hamster ovary (CHO) cells in single-use polycarbonate shake flasks with online monitoring of the oxygen transfer rate (OTR). OTR in triplicate (open orange squares, open pink triangles, open black circles) over the cultivation time. Closed black arrow indicates decrease in OTR upon depletion of glucose and lactate, open arrow indicates “shoulder” in OTR. Data from experiment 2 (Supplementary Table S1) with off-set (Supplementary Figure S4) subtracted.

A cut-off phase of 8 min was suited for both flask types to enable linear fitting of the oxygen partial pressure decrease over time (orange parts in Supplementary Figures S6, S7, respectively). However, the calculated slope (m) for the single-use polycarbonate flask (Supplementary Figure S7, m = −0.449 mV h^−1^) was higher compared to the glass flask (Supplementary Figure S6, m = −0.362 mV h^−1^) as the water vapor equilibrium was not reached (see Supplementary Data *Influence of Humidity* for a detailed discussion). Consequently, the oxygen partial pressure in the polycarbonate flask decreased because of the breathing activity of the cells and by “dilution” with water vapor, resulting in a higher slope compared to the glass flask. However, as the off-set remained constant at the end of the cultivation (Supplementary Figure S4), it was subtracted from each measurement point (Figure 4). The course of the corrected OTR showed the same drop and shoulder (Figure 4, closed and open arrow, respectively) that was observed in glass flasks (Figure 3).

**FIGURE 5:**
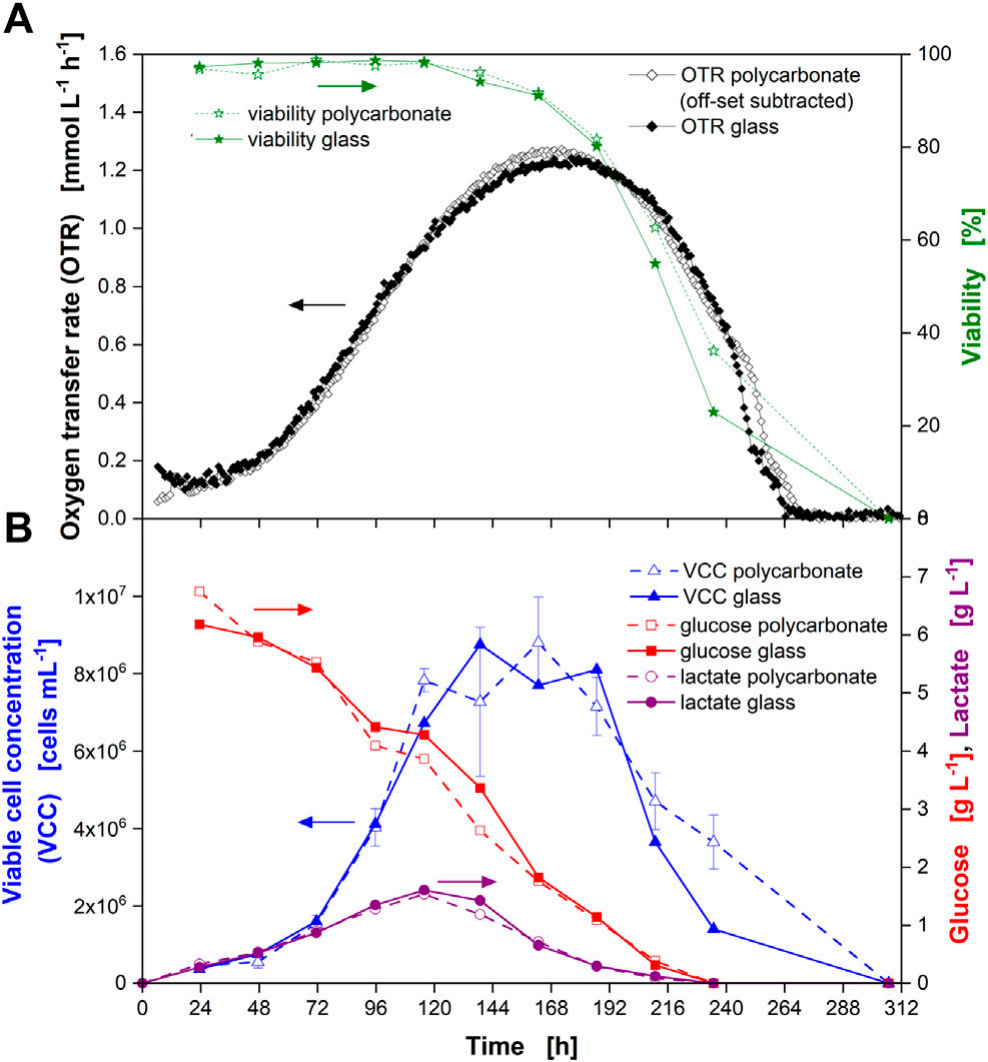
Comparison of Chinese hamster ovary (CHO) culture behavior in glass and single-use polycarbonate shake flasks with online monitoring of the oxygen transfer rate (OTR) and manual (“offline”) sampling. **(A)** Averaged OTR from triplicate measurement for polycarbonate flasks (open black diamonds) and glass flasks (closed black diamonds) as well as culture viability in polycarbonate flask (open green stars) and glass flask (closed green stars) are depicted over the cultivation time. **(B)** Viable cell concentration (VCC) in polycarbonate (open blue triangles) and glass (closed blue triangles) flasks, glucose concentration in polycarbonate (open red squares) and glass (closed red squares) flasks as well as lactate concentration in polycarbonate (open purple circles) and glass (closed purple circles) flasks over the cultivation time. Data from experiment 2 (see Supplementary Table S1). Single measurements of the OTR are depicted in Figure 3 (glass flasks) and Figure 4 (polycarbonate flasks), respectively.

The culture behavior in glass and single-use polycarbonate flasks was compared by analysing the online and offline data (Figure 5). Figure 5 depicts the data for glass flasks already presented in Figure 3 and the OTR data for single-use polycarbonate flasks also shown in Figure 4.

As demonstrated above, the mass transfer resistance was similar for the cotton plug and the vent-cap. In addition, the k_L_a values for gas-liquid mass transfer were in the same order of magnitude and the maximum oxygen transfer capacity (OTR_max_) achievable under the chosen cultivation conditions of about 7.2 mmol L^−1^ h^−1^ was much higher than the OUR of the culture. Consequently, a similar culture behavior was expected. This similar culture behavior was confirmed by the similar course of the OTR (Figure 5A, diamonds). The similar culture behavior was further proven by the similar course of the offline measured parameters (Figure 5A, stars and Figure 5B). Further, it was confirmed that potential leakage of bisphenol A from the polycarbonate flask did not affect any of the parameters determined for the CHO cell line used in this study.

### Culture Reproducibility and Cultivation in a Different Medium

**FIGURE 6:**
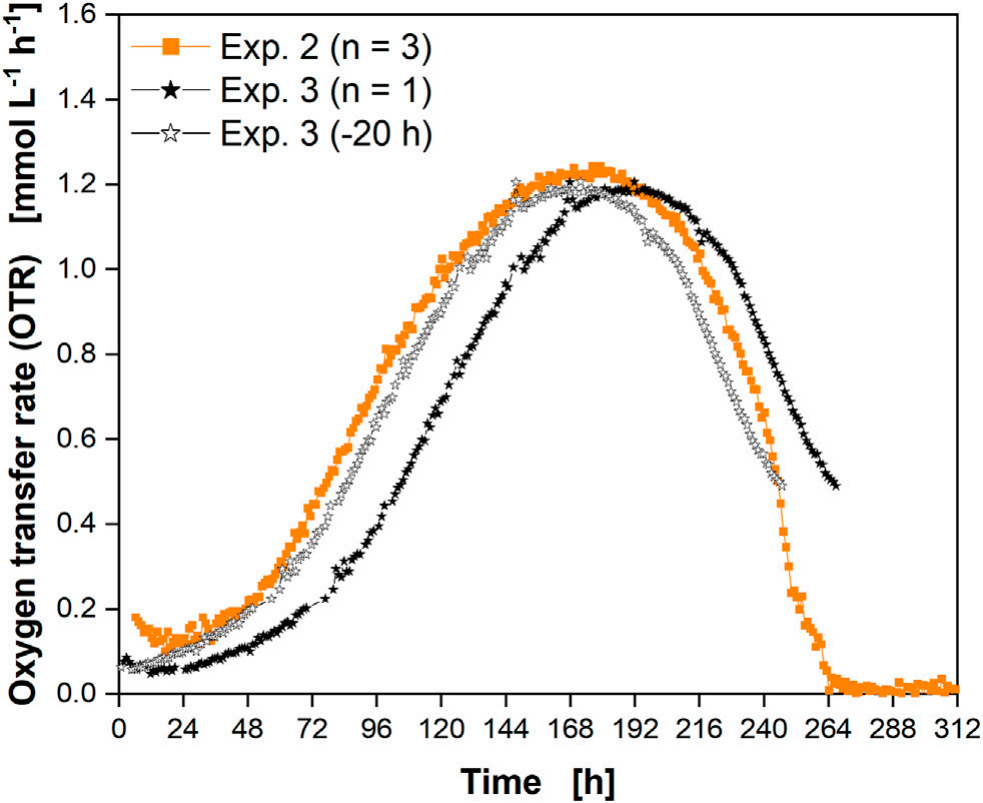
Reproducibility of culture behavior in glass flasks. The OTR is shown as the average of a triplicate determination (closed orange squares, experiment 2), and as a single determination (experiment 3, closed black stars). Data from experiment 3 were shifted by 20 h (open black stars) for comparison with experiment 2. Compare Supplementary Table S1 for experiment comparison.

The reproducibility of the culture behavior and the OTR signal was determined by a third cultivation using glass flasks (Figure 6). The course of the OTR from the second experiment (Supplementary Table S1) in glass flasks (Figure 3) is shown for comparison.

Both experiments were inoculated from the same cryogenic vial with 6 weeks’ time lag between the experiments. A difference in the lag-phase was visible between the cultures (Figure 6A, closed orange squares and closed black stars). This difference could be due to the use of cells from different passage numbers (Supplementary Table S1). Differences in the lag phase could also be the cellular response to different times of harvesting the preculture. As a result, the cells would be in a different growth phase (e.g., early instead of mid-exponential growth phase). Except for these minor differences, the overall respiration profiles matched well. Considering the shift of ∼20 h (open black stars) corresponding to the cell doubling time, the OTR data from the third experiment (closed black stars) aligned well between the two consecutive experiments. This confirmed that the culture behavior of the CHO cells was highly reproducible and that monitoring of the OTR was suited to reveal differences in the lag-phase. Thus, measurement of the OTR facilitates the adaptation of sampling points to the progress of the culture to match and to compare consecutive experiments. In addition, cell culture monitoring during passaging could be carried out in the future to ensure that the cells are in exactly the same growth phase upon transfer without the need for manual sampling.

**FIGURE 7:**
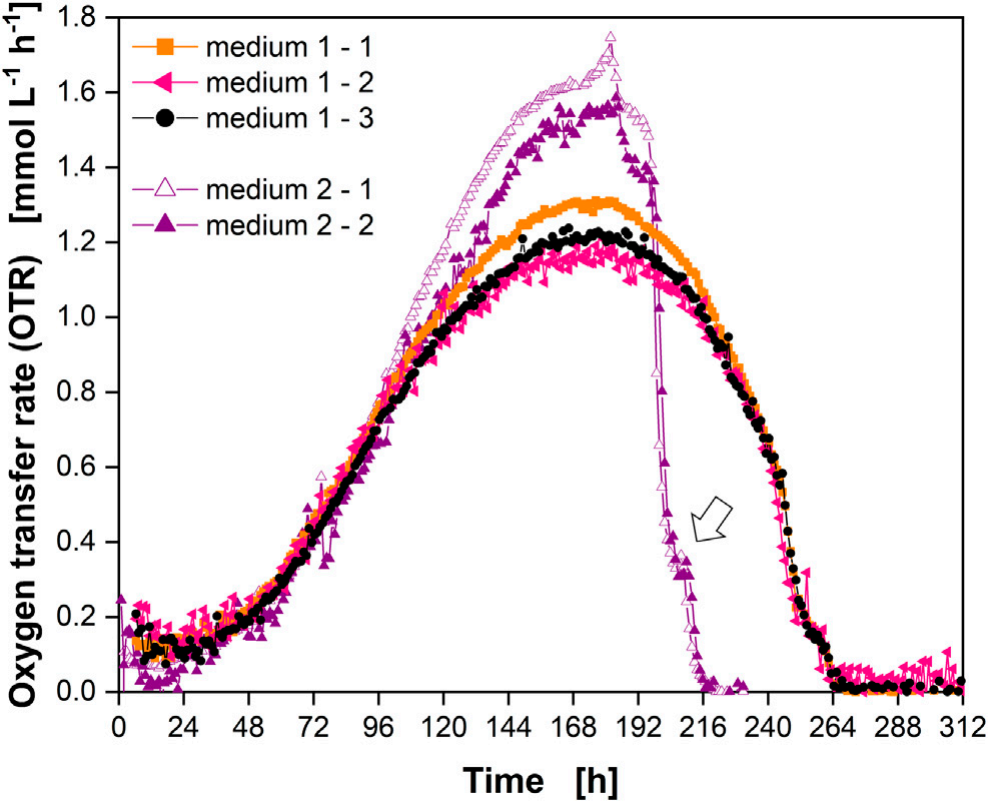
Comparison of Chinese hamster ovary (CHO) culture behavior in different media. OTR in triplicate for PowerCHO^TM^ two medium (“medium 1”) (closed orange squares, closed pink triangles, closed black circles) and in duplicate for EX-CELL® Advanced™ CHO Fed-batch Medium (“medium 2”) (open and closed purple triangles). Open arrow indicates “shoulder” in OTR. Data for medium one from experiment 2 (Figure 3) (see Supplementary Table S1). Data for medium two from experiment 4 (Supplementary Table S1).

Cells were cultured in a different medium in a fourth experiment (Supplementary Table S1) to demonstrate the future application potential of the RAMOS technology for mammalian cell cultivation. A batch cultivation was carried out in EX-CELL® Advanced™ CHO Fed-batch Medium (“medium 2”) (Figure 7, purple triangles). Data for cultivation in PowerCHO^TM^ two medium (“medium 1”) from Figure 3 are shown for comparison (Figure 7, orange squares, black circles, pink triangles).

The course of the OTR clearly differed between the two media. The end of active respiration was reached about 2 days earlier for medium 2 and higher OTRs were reached during cultivation. Similar to medium 1 a “shoulder” was also observed for medium 2 (open arrow). These data demonstrated that the application of RAMOS for an initial comparison of culture media was possible and enabled a first comparison of the overall culture behavior and the peak maximum OTR (in terms of value and timepoint) without sampling. In the future, correlation with offline parameters will be investigated in detail. If these correlations (e.g., cell specific oxygen uptake rate) are already known for a cell line, direct calculation of derived cellular and metabolic information (e.g., VCC) is possible.

## Conclusion

The data presented in this study demonstrated the benefit of time-resolved monitoring of the OTR to increase the information content of cultivations of CHO cells in shake flasks. By monitoring the OTR, information about the culture behavior could non-invasively be obtained without manual sampling. The data amount was increased 24-fold compared to manual sampling, which was commonly carried out once per day. Parallelized monitoring was possible in glass and single-use polycarbonate shake flasks. In comparison to fully instrumented bioreactors, easier parallelization and utilization of lower filling volumes is possible, while simultaneously obtaining comparable results.

Determination of the mass transfer resistance of two different sterile closures (cotton plugs and vent-caps) showed only a minor influence on the total oxygen mass transfer in shake flasks. Values determined were used to adjust the gassing rate of the RAMOS device. Consequently, the culture behavior in actively aerated, monitored flasks was shown to be identical to flasks stoppered with cotton plugs.

The reproducibility of the OTR data within one experiment exhibited a standard deviation of less than 10% for both flask types. Monitoring the OTR with the RAMOS device is non-invasive and requires no manual intervention and no process-interfering volume sampling. Thus, compared to conventional sampling, it is clearly less labor-intensive, more flexible, and does not affect culture behavior. It was demonstrated that the OTR was very close to the OUR under the conditions applied in this study. The course of the OTR reflected the course of the VCC until the maximum OTR was reached. In addition, the depletion of all carbon sources and the end of active respiration was reflected in the OTR as it dropped to zero. Furthermore, the respiration profiles and the cell growth in glass and single-use polycarbonate flasks were shown to be identical, if the raw data was reasonably treated. Comparison with a second medium demonstrated the potential of the technology to reveal differences in the OTR in different culture media. This way, a valid comparison of satellite cultures in industrial production processes which use downscale culture models with culture media that can be slightly different from those used in the large bioprocess reactors might be possible. This is especially advantageous, if correlations between the OUR and other culture parameters are known.

In the future, it will be interesting to apply monitoring of the respiration activity to 1) more thoroughly screen media performance, 2) estimate parameters like glucose concentration and viable cell concentration without sampling, and 3) spot changes in culture performance (e.g., changes in lag-phase) at early stages of process development (at small scale).

## Data Availability

The original contributions presented in the study are included in the article/Supplementary Material, further inquiries can be directed to the corresponding author.
