# Supplementary material for: Time-Resolved Monitoring of the Oxygen Transfer Rate of Chinese Hamster Ovary Cells Provides Insights Into Culture Behavior in Shake Flasks
Source: Front Bioeng Biotechnol. 2021 Aug 25;9:725498. doi: 10.3389/fbioe.2021.725498 (PMC8423908; doi:10.3389/fbioe.2021.725498)
Supplement: Supplementary file 1 [file DataSheet1.docx]

Supplementary Material

**Supplementary Table 1.** Overview of experiments presented in this study.

|  | Experiment 1 | Experiment 2 | Experiment 3 | Experiment 4 |
| --- | --- | --- | --- | --- |
| Figures | Figure 2 | Figures 3-5, Supp. Figures 2-4,6,7 | Figure 6 | Figure 7 |
| Culture Medium | PowerCHO^TM^ 2 (“medium 1”) | PowerCHO^TM^ 2 (“medium 1”) | PowerCHO^TM^ 2 (“medium 1”) | EX-CELL® Advanced™ CHO Fed-batch Medium (“medium 2”) |
| Media Supplements | 6 mM glutamine, 1% PenStrep | 6 mM glutamine, 30 µg mL^-1^ kanamycine | 6 mM glutamine, 1% PenStrep | 6 mM glutamine, 1% PenStrep |
| Number of passages after thawing | 7 | 2 | 14 | 7 (including adaptation from medium 1 to 2) |
| Number of cryogenic vial | 1 | 2 | 2 | 3 |

**Supplementary Table 2.** Comparison of glass and polycarbonate flasks.

| Parameter | Glass flask | Polycarbonate flask |
| --- | --- | --- |
| Liquid film formation* | Yes | Less |
| Surface properties | Hydrophilic, ionic | Hydrophobic, non-ionic |
| Sterile closure | Wrapped cotton plug | Vent-cap |
| Carbon footprint | Low (multi-use) | High (single-use) |
| Gassing in RAMOS | Directly into the headspace of the flask | Indirectly above the vent-cap membrane |

*****A liquid film is distributed on the flask wall as the liquid rotates inside the flask. This liquid film largely contributes to the oxygen transfer capacity in a shake flask. With increasing hydrophobicity, liquid film formation is reduced (Maier & Büchs, 2001).

## Determination of mass transfer characteristics for glass and single-use polycarbonate flasks:

First, the water evaporation rate from the glass flasks was determined under the experimental conditions of the "offline" flasks (see Section 2.1). Next, the model for calculation of the mass transfer resistance developed by Mrotzek, Anderlei, Henzler, and Büchs (2001) based on the model of Henzler and Schedel (1991) was used to determine the diffusion coefficient of carbon dioxide (DCO_2_) and the average diffusion coefficient of oxygen (DO_2_) over the plug height (Supplementary Figure 1, left). DO_2_ was then used to calculate the mass transfer resistance of the plug for oxygen (k_st,O2_). Values used for the calculation are given in Supplementary Table 2.

The DCO_2_ of the used cotton plugs was determined to be 0.073 cm² s^-1^ under the applied experimental conditions. This DCO_2_ is in the same range as the DCO_2_ values previously determined for cotton plugs (~0.10 cm² s^-1^; depending on the bulk density) (Anderlei et al., 2007; Mrotzek et al., 2001). The average DO_2_ was calculated to 0.095 cm² s^-1^ resulting in a k_st,O2_ of 0.81∙10^-5^ mol s^-1^ (Supplementary Figure 1, left).

**

**

**Supplementary Figure 1: Workflow for determining the mass transfer coefficient of the cotton plug and vent-cap (k_st,O2_).** Values and units for parameters are given in Supplementary Table 1. Left: Approach for glass flasks stoppered with cotton plugs. The model used is described in-depth by Anderlei et al. (2007). It calculates the diffusion coefficient for carbon dioxide (DCO_2_) and average oxygen diffusion coefficient over the plug height (avg. DO_2_). Calculation of oxygen mass transfer resistance (k_st,O2_) with DO_2_ and molar gas volume (Vmo). Right: Approach for calculation of k_st,O2_ for polycarbonate flasks with vent-cap modified from Sieben, Giese, Grosch, Kaufmann, and Büchs (2016). Mass transfer resistance for water vapor (k_st,H2O_) is calculated from water evaporation (J_H2O_) and partial pressure difference between outside (p_H2O,out_) and inside (p_H2O,in_). The membrane in the vent-cap resembles a sealing foil in its characteristics rather than a plug. Consequently, the model by Mrotzek et al. (2001) used for cotton plugs was not suitable. Instead, the approach presented by Sieben et al. (2016) for characterization of the mass transfer in microtiter plate sealing foils was used (Supplementary Figure 1, right side). It was assumed that the membrane was not selective for any of the gases. The evaporation rate of water from the flask was determined experimentally and used to calculate k_st,H2O_ (Supplementary Figure 1, right side). The water vapor outside the flask (p_H2O, outside_) was adjusted by the set-point of the relative humidity (RH) in the incubator (RH = 70%). Inside the flask, fully saturated conditions (RH = 100%) were assumed. This assumption was previously proven experimentally (Sieben et al., 2016). It was also assumed that the whole membrane area was available for diffusion. Finally, k_st,O2_ was calculated to be 1.17∙10^‑5^ mol s^-1^.

**Supplementary Table 3.** Explanation of variables and values used for calculation of the mass transfer resistances of different sterile closures at a temperature of 36.5°C.

| Variable | Meaning | Value | Unit |
| --- | --- | --- | --- |
| A | Area of sterile closure | 7.07 (cotton plug)  1.26 (vent-cap membrane) | cm² |
| Avg. DO_2_ | Average diffusion coefficient over the plug height | 0.095 (cotton plug) | cm² s^-1^ |
| DCO_2_ | Diffusion coefficient of carbon dioxide | 0.073 (cotton plug) | cm² s^-1^ |
| $\frac{D_{eff,H2O}}{H}$ | Effective diffusion coefficient of water vapor in relation to height | 0.287 (vent-cap) | cm s^-1^ |
| $\frac{D_{eff,O2}}{H}$ | Effective diffusion coefficient of oxygen in relation to height | 0.235 (vent-cap) | cm s^-1^ |
| H | Height of sterile closure inside the flask neck | 3.25 (cotton plug)  Unknown (vent-cap) | cm |
| J_H2O_ | Water evaporation rate from flask | 0.012 (glass flask stoppered with cotton plug)  0.017 (polycarbonate flask closed with vent-cap) | g h^-1^ |
| k_L_a | $k_{L}a=\frac{\mathrm{OTR}_{\max}}{L_{O2}\cdot p_{\mathrm{abs}}{\cdot y}_{O2,out}}$ | 42 | h^-1^ |
| k_st,O2_ | Oxygen mass transfer coefficient of the sterile closure | 0.81∙10^-5^ (cotton plug)  1.17∙10^-5^ (vent-cap) | mol s^-1^ |
| L_O2_ | Oxygen solubility in culture medium | 0.000934 | mol L^-1^ bar^-1^ |
| OTR_max_ | Maximum oxygen transfer rate at given cultivation conditions (70% RH, 5% CO_2_) | 7.11 (cotton plug)  7.26 (vent-cap)  7.50 (gas-liquid only) | mmol L^-1^ h^-1^ |
| p_abs_ | Absolute pressure | 1.013 | bar |
| p_H2O,in_ | Water vapor partial pressure in headspace (100% RH) | 0.06120 | bar |
| p_H2O,out_ | Water vapor partial pressure in incubator (70% RH) | 0.04824 | bar |
| R | Universal gas constant | 0.083 | bar L mol^-1^ K^‑1^ |
| RH_in_ | Relative humidity inside the shake flask | 100 | % |
| RH_out_ | Relative humidity set-point of the incubator | 70 | % |
| T | Temperature | 36.5 | °C |
| V_G_ | Gas volume of the shake flask | 0.24 – 0.26 | L |
| V_L_ | Liquid volume in shake flask | 0.05 | L |
| Vmo | Molar gas volume at 36.5°C | 25,410 | cm³ mol^-1^ |
| y_O2,liquid_ | Molar fraction of oxygen in the liquid | 0 | - |
| y_O2,outside_ | Molar fraction of oxygen in incubator (70% RH, 5% CO_2_) | 0.191 | - |

**

**

**Supplementary Figure 2:** 1^st^ derivative of the average OTR in glass flasks. Data from experiment 2 (see Table 2) are also depicted in Figure 4.





**Supplementary Figure 3: Course of pH and osmolality for a cultivation of Chinese hamster ovary (CHO) cells in shake flasks.** Course of the pH (open blue squares) and the osmolality (closed orange circles) over the cultivation time. CHO cells (initial VCC = 0.2‧10^6^ mL^-1^) were cultured at 36.5°C in serum-free chemically defined medium (PowerCHO^TM^ 2) supplemented with 6 mM glutamine and 30 µg mL^-1^ kanamycin. Flasks were passively ventilated by a cotton plug in an incubator environment with 5% CO_2_ in synthetic air at 70% relative humidity. Cultivation conditions: 250 mL glass shake flasks, 50 mL filling volume, 140 rpm shaking speed, 50 mm shaking diameter. Data from experiment 2, which is shown in Figure 4.

**

**

**Supplementary Figure 4: Cultivation of Chinese hamster ovary (CHO) cells in single-use polycarbonate shake flasks with online monitoring of the oxygen transfer rate (OTR).** OTR in triplicate (open orange squares, open pink triangles, open black circles) over the cultivation time. CHO cells (initial VCC = 0.2‧10^6^ mL^-1^) were cultured at 36.5°C in serum-free chemically defined medium supplemented (PowerCHO^TM^ 2) with 6 mM glutamine and 30 µg mL^-1^ kanamycin. The off-set, most probably caused by water vapor diffusion from the headspace of the flask through the membrane in the vent-cap into the gap between membrane and oxygen sensor, is not yet subtracted here. Flasks were gassed above the vent-cap membrane with 5% CO_2_ in synthetic air at a rate of 6.25 mL min^-1^ (online) and passively ventilated by a vent-cap in an incubator environment with 5% CO_2_ in air at 70% relative humidity (offline). Cultivation conditions: 250 mL single-use polycarbonate shake flasks, 50 mL filling volume, 140 rpm shaking speed, 50 mm shaking diameter. Data from experiment 2.

## Influence of humidity:

The aeration of online monitored polycarbonate flasks took place above the vent-cap (Figure 1B). Consequently, the oxygen sensor was separated from the headspace by the membrane in the vent-cap and by the small gap needed for gassing (Figure 1B). This gap had a gas volume of approximately 3 mL and was gassed with dry air from a gas bottle during the gas flow phase. As soon as the valves were closed and the measurement phase started, there still was dry air above the vent-cap membrane, but gas fully saturated with water vapor inside the flask. Consequently, it is assumed that water vapor diffusion from the inside of the flask into the adapter across the vent-cap membrane took place. This water vapor "diluted" the oxygen concentration in the gassing gap of the adapter. As a result, a decrease in the oxygen partial pressure in the gassing gap of the adapter over time was measured by the oxygen sensor that erroneously resulted in the calculation of a constant positive OTR (Figure 3A, closed black squares).





**Supplementary Figure 5: Measurement in the adapted RAMOS device using only water (non-biological system).** (A) OTR over time in single-use polycarbonate flask (closed black squares) and glass flask (closed grey triangles) filled only with deionized water without biology. (B) Raw sensor voltage signal of the electrochemical oxygen sensor between 5.2 and 6.4 hours in single-use polycarbonate flask (closed black squares) and glass flask (closed grey triangles). Only every 10^th^ data point is shown. For the polycarbonate flask, arrow marks the beginning of the measurement phase (20 min) including a cut-off-phase of 8 min (open blue squares). Directly after the measurement phase, before the next gas flow phase (40 min) starts, the high-flow phase is entered to refresh the gas atmosphere in the gassing gap of the adapter between the membrane in the vent cap and the oxygen sensor, resulting in a rapid increase of the sensor voltage signal. Cultivation conditions: 250 mL flasks, 50 mL filling volume, 140 rpm shaking speed, 50 mm shaking diameter, temperature of 36.5°C.

The hypothesis of water vapor influencing the oxygen measurement was supported by the sensor voltage signals from the two different flask types (Supplementary Figure 5B). For the glass flask, the sensor voltage remained constant over time (Supplementary Figure 5B, grey triangles). A constant sensor voltage was expected, as no changes in water vapor and no oxygen consumption occurred in this non-biological system. However, the sensor signal for the polycarbonate flask significantly decreased after the beginning of the measurement phase (Supplementary Figure 5B, black squares, arrow). From the shape of the decrease, a concentration-dependent effect was likely, because the decrease was not linear. The actual value of the erroneously calculated OTR depends on the length of the so-called “cut-off phase”. In this phase, the valves (see Figure 1A) are already closed, but the decrease in partial pressure has not yet reached a steady pace and is, therefore, not considered for calculating the OTR. In the measurement setting depicted in Supplementary Figure 5, the duration of the cut-off phase was set to 8 min (Supplementary Figure 5B, blue symbols). This means that the first 8 min of the measurement phase were not considered for calculation of the OTR. For microbial cultivations carried out in glass flasks, the duration of the cut-off phase is typically 1 - 2 min. After the first 8 min of the measurement phase, the sensor voltage signal of the polycarbonate flasks filled with water had reached 85% of the value reached at the end of the measurement phase (Supplementary Figure 5B). However, as the equilibrium still was not reached and the sensor signal decreased further, linear fitting of the sensor voltage signal in the remaining part of the measurement phase resulted in a subsequent calculation of an OTR of about 0.55 mmol L^-1^ h^-1^. With increasing cut-off time the absolute value of the OTR off-set will decrease as the water vapor in the gassing gap of the adapter further continues to approach an equilibrium. As a result, a cut-off time of 8 min was chosen as a compromise between the absolute value of the OTR off-set and the overall duration of the measurement phase.

**

**

**Supplementary Figure 6: Course of the signal of the electrochemical oxygen sensor at the highest breathing activity.** Sensor voltage signal of electrochemical sensor from glass flask 1 (closed orange squares in Figure 4) after 176 hours. m values represents the slope from linear fit for measurement phase excluding cut-off phase (orange symbols, green line). Arrow marks the transition from cut-off phase (8 min) to the part of the measurement phase that is used for evaluation of the sensor voltage decrease (12 min). The total duration of the measurement phase was set to 20 min.





**Supplementary Figure 7: Course of the signal of the electrochemical oxygen sensor at the highest breathing activity.** Sensor voltage signal of the electrochemical sensor from single-use polycarbonate flask 1 (open orange squares in Supplementary Figure 5) after 176 hours. m value represents the slope from linear fit (green line) for measurement phase without cut-off phase (orange). Arrow marks the transition from cut-off phase (8 min) to the part of the measurement phase that is used for evaluation of the sensor voltage decrease (12 min). The total duration of the measurement phase was set to 20 min.

**References**

Anderlei, T., Mrotzek, C., Bartsch, S., Amoabediny, G., Peter, C. P., & Büchs, J. (2007). New method to determine the mass transfer resistance of sterile closures for shaken bioreactors. *Biotechnology and Bioengineering, 98*(5), 999-1007. doi:10.1002/bit.21490

Henzler, H. J., & Schedel, M. (1991). Suitability of the shaking flask for oxygen-supply to microbiological cultures. *Bioprocess Engineering, 7*(3), 123-131. doi:10.1007/Bf00369423

Maier, U., & Büchs, J. (2001). Characterisation of the gas-liquid mass transfer in shaking bioreactors. *Biochemical Engineering Journal, 7*(2), 99-106. doi:10.1016/S1369-703x(00)00107-8

Mrotzek, C., Anderlei, T., Henzler, H. J., & Büchs, J. (2001). Mass transfer resistance of sterile plugs in shaking bioreactors. *Biochemical Engineering Journal, 7*(2), 107-112. doi:10.1016/S1369-703x(00)00108-X

Mühlmann, M. J., Forsten, E., Noack, S., & Büchs, J. (2018). Prediction of recombinant protein production by *Escherichia coli* derived online from indicators of metabolic burden. *Biotechnology Progress, 34*(6), 1543-1552. doi:10.1002/btpr.2704

Sieben, M., Giese, H., Grosch, J. H., Kaufmann, K., & Büchs, J. (2016). Permeability of currently available microtiter plate sealing tapes fail to fulfil the requirements for aerobic microbial cultivation. *Biotechnology Journal, 11*(12), 1525-1538. doi:10.1002/biot.201600054

Truesdale, G. A., Downing, A. L., & Lowden, G. F. (1955). The solubility of oxygen in pure water and sea-water. *Journal of Applied Chemistry, 5*(2), 53-62. doi:10.1002/jctb.5010050201
